# Comparison between Ag (I) and Ni (II) removal from synthetic nuclear power plant coolant water by iron oxide nanoparticles

**DOI:** 10.1186/2052-336X-11-21

**Published:** 2013-07-25

**Authors:** Mohammad Hossein Salmani, Mohammad Hassan Ehrampoush, Mohaddeseh Aboueian -Jahromi, Mohsen Askarishahi

**Affiliations:** 1 Department of Environmental Health Engineering, Faculty of Public Health, Shahid Sadoughi University of Medical Sciences, Yazd, Iran

**Keywords:** Nanoparticles iron oxide, Nickel, Silver, Removal, Synthetic coolant

## Abstract

The impact of effective parameters such as iron oxide nanoparticles dosage, contact time and solution pH was optimized for removal of Ag(I) and Ni(II) in the nuclear cooling system and the best conditions were compared**.** Nearly complete removal (97%) of Ni(II) and Ag(I) were obtained at adsorbent dosage of 40 and 20 g/L, respectively. Experiments showed that 4 hours was a good choice as optimum contact time for two ions removal. The effective parameter was pH, so that maximum removal efficiency was obtained for Ag(I) in acidic pH=3 and for Ni(II) in basic pH=10. It seems that removal of Ag(I) was controlled by adsorption-reduction mechanism, but Ni(II) could place only adsorption. Langmuir and Freundlich model was more suitable for nickel and silver removal by this adsorbent, respectively. Ag(I) and Ni(II) removal efficiency trend by this adsorbent is similar at periods but different in the concentrations, pHs and equilibrium model. The obtained results were very promising, as both Ag(I) and Ni(II) were effectively removed from synthetic wastewater and there was a possibility to remove Ag(I) very fast. Hence, the idea of using nanoparticles for application of metal ions removal from wastewaters seems to be very efficient and quite promising.

## Introduction

The primary coolant is an essential cooling medium used to control heat in a nuclear power plant. The most commonly used primary coolant is high-purity water. During the operation of a nuclear power plant, corrosion products are released from the surfaces of the primary circuits into the cooling system. The corrosion products originate from the internal surfaces of piping and steam generator under the condition of high pressure and temperature. Some corrosion species are activated by the neutron flux in the reactor core and various forms of radionuclides are produced [1]. The important radionuclides ^60^Co and ^58^Co are present in liquid waters that released from pressurized water nuclear power reactors. The ^58^Co can be formed by fast neutron capture of ^58^Ni. Various forms of radionuclides cause activity build-up, contamination of the primary coolant system and occupational radiation exposure. Therefore, the system decontamination is an important task in a nuclear power plant by reducing the radiation and metal concentration level. To achieve this goal, an alternative process is required to remove corrosion products [2].

Various physicochemical methods have been employed for removal of heavy metals from waste discharges, which include chemical precipitation, flotation, filtration, extraction, ion-exchange, electrochemical operation and adsorption process [3-6]. Ni(II) removal from synthetic nuclear power plant coolant water has been studied by such different methods as ion exchange resins (IRN77 and SKN1), stack configuration of continuous Electro-deionization, coir pith, magnetic filter – electro-deionization hybrid separation system [7-9]. The used conventional techniques for removal of chemical contaminants from wastewaters are adsorption process. These are mainly preferred when the enrichment of trace metal amounts or a high selectivity for a certain metal are required. Adsorption of heavy metals on mineral adsorbents such as Iron oxides and zero-valant iron has been widely studied by researchers. In the recent works, efforts are being made to use nanomaterials because nonomaterials have higher surface area and greater active sites for interaction with metal species [4,10-12]. As a result, the use of nanoparticles for contaminant removal is more effective than the mass.

Besides lots of advantages of zero-valent Iron nanoparticles (nZVI), one of its major drawbacks is its sensitivity to oxidation in aqueous solution. In the recent years, the iron oxide nanoparticles have been investigated for the removal of organic and inorganic contaminants. In this study, iron oxide nanoparticle, a new and extensively usable material, was investigated for removal of Ag(I) and Ni(II) from synthetic nuclear power plant coolant water. The aim of this study was to compare Ag(I) and Ni(II) removal based on adsorption by iron oxide nanoparticles that was developed a shell layer iron oxide and a core zero-valent iron.

## Materials and methods

Iron oxide nanoparticles can be prepared by oxidation of nZVI with oxygen [13]. The properties of used nZVI as reported by the suppliers are shown in Table 1.

**Table 1:** Physical and chemical property of zero-valent iron nanoparticles

**Physical property**	
Average particle size	< 30 nm
Specific surface area	> 20 m^2^/g
Bulk density	0.04 ~ 5 g/cm^3^
**Chemical component**	
O	<0.1%
Impurity	<0.3(International standard 0.4)
Fe	Surplus

So, iron oxide nanoparticles were synthesized by exposing nZVI to air and as a result ignition and heat were produced. In order to homogenize the particles, they were grinded following cooling down. It is documented in the literature that the concentrations of heavy metals in nuclear power plant coolant water vary by several orders of magnitude [1,2,14]. Attempt was made to use realistic concentration ranges so, the composition of this solution was Sb(V), Co(II), Fe(III), Ni(II), Ag(I), B(III), Cr(III), Li(I), Cs(I) with concentration of 5, 1, 30, 15, 5, 20, 4, 0.5, 0.5 mg/L, respectively. The salts of Sb_2_O_5_, Co(NO_3_)_2_.6H_2_O, Fe(NO_3_)_3_.9H_2_O, Ni(NO_3_)_2_.6H_2_O, AgNO_3_, H_3_BO_3_, Cr(NO_3_)_3_.9H_2_O, Li(OH).H_2_O, CsNO_3_ of analytical grade (Merck) were used. Stock and diluted solution were prepared by dissolving salts in distillated water. pH adjustment was performed with 0.1 N HCl and NaOH solution. All experiments were performed in environmental chemistry laboratory.

In order to determine the size of nanoparticles in solution, a Scanning Tunneling Microscopy (STM), SS_2_ model (Iran) was used. A Varian model 20AA atomic absorption spectrometer was applied for measurement of ion concentrations in solution. Chemical substances were weighed at precision of ±0.0001 g with Mettler model digital laboratory scale. Solution pH was measured by a Sension 3 model digital pH-meter.

### Batch adsorption experiments

After producing iron oxide nanoparticles in solution, their efficiency in Ni(II) and Ag(I) removal from synthetic nuclear power plant coolant water was assessed by several experiments. The experiments were conducted with variable factors such as pH, contact time and nanoparticle dosage. All experiments were performed in 250 ml flask in batch system at laboratory temperature. After reaction was completed, the suspension was filtered on 0.2 μ cellulose filter, centrifuged at 3500 rpm and then supernatant solution was stabilized with 1% (v/v) nitric acid. Concentrations of Ni(II) and Ag(I) were determined with an atomic absorption spectrometer. In order to ascertain the results, the atomic absorption spectrophotometer was arranged in triplicate mode and the mean concentration was used to calculate the parameters. The experimental conditions investigated are shown in Table 2.

**Table 2:** Experimental conditions investigated

**Parameter**	**Values investigated**
Contact time, h	0.17, 0.5, 1, 4, 8 and 24
Iron oxide nanoparticle dosage g/ml	0.5, 1, 10, 15, 25, 30 and 40
Initial concentration of Ni, mg/L	15
Initial concentration of Ag, mg/L	5
pH of the aqueous solution	2, 3, 4, 5, 6, 7, 8, 9 and 10
Temperature, °K	300
Iron oxide nanoparticle size, nm	40

### Data analysis

The mean equilibrium concentration of metal ions from output atomic absorption (C_e_), time (t), pH and amount of adsorbed ions on the adsorbent at any time (q) was saved in data file of SPSS. Removal efficiency (R), t/q, q_e_ and C_e_/q_e_ for all cases were calculated by formulate in SPSS and then saved. In order to examine the control of adsorption mechanism and kinetic model, the experimental data were used to test. For obtaining the best adsorption equilibrium, some different isotherm equations were tested and the (C_e_/q_e_) versus C_e_ was plotted finally. The value of Q^0^ and b was determined from slope and interception of the obtained straight line. Three different models were tested for obtaining the best kinetic model. Finally, the t/q versus t gave a straight line and then q_e_ and k_2,ads_ were determined from the slope and interception of the plotting, respectively. It is important to notice that the experimental estimation of q_e_ is not necessary for the application of this model.

## Results and discussion

### Characterization of iron oxide nanoparticle

**Figure 1:**
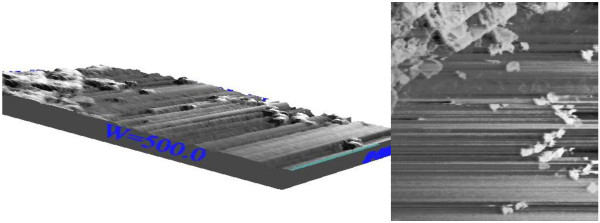
STM images of iron oxide nanoparticles.

It is still a big challenge to develop simple and reliable synthetic methods for hierarchically self-assembled architectures with designed chemical components and controlled morphologies, which strongly affect the properties of nanomaterials [15]. As is known, the exposure of nZVI to oxygen results in the development of an iron oxide layer, leading to a core-shell structure of the iron nanoparticle in which the core preserves the nZVI nature, while the shell contains iron oxides (Fe_2_O_3_, Fe_3_O_4_, FeOOH) [16]. Scanning Tunneling Microscopy (STM) is a widely used technique for the determination of morphology and size distribution of prepared particles in the scales of micro to nano range [17]. Figure 1 depicts STM of the iron oxide nanoparticle. It shows that these particles are nano and their size is less than 40 nm. After oxidation process, the oxidized nanoparticle had increased in size for particles about 10 nm.

A literature survey suggests that the precise composition of the oxide shell depends on the fabrication processes and also on environmental conditions. For example, if we heated the obtained Fe_3_O_4_ at 250°C in air for 5 h, the color changed from black to red-brown [16]. The X-ray diffraction (XRD) pattern of the red-brown product was very close to that of the Fe_2_O_3_ and agreed well with the standard XRD pattern of γ-Fe_2_O_3_[15]. With respect to production method and color (red-brown) of nanoparticles, the prepared nanoparticles were γ-Fe_2_O_3._

nZVI have some disadvantages including: a) Its mechanism in removing some metals like Ni(II) is both reduction and adsorption in which iron ions enter to environment due to reduction process while its permitted limit is 3 mg/L. b) different forms of nZVI (i.e. fresh, aged, and surface modified) are differentially toxic to rodent nerve cells [18]. So, in this study nZVI was oxidized, in which oxide layer thickness, and as a result absorption process is increased and its operation and maintenance are more comfortable.

### Effect of time on Ni(II) and Ag(I) removal

**Figure 2:**
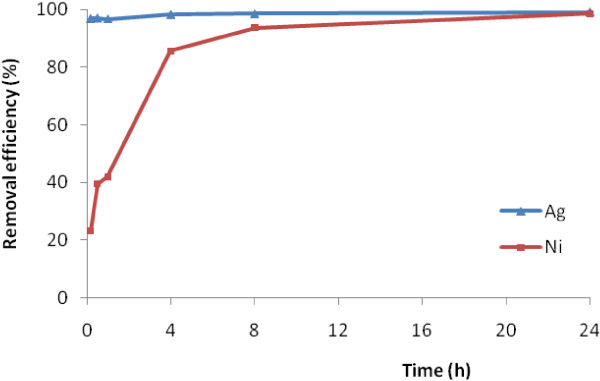
Effect of time on Ni(II) and Ag(I) removal at pH of 2.7 and 35 g/L adsorbent dosage.

Figure 2 predicted variation experiments that were performed with the same terms in different times.

According to Figure 2, there is a break point at after 4 h contact time. So, 4 h can be chosen as optimum time for both ions. Efecan Nazlı concluded that equilibrium time for Ni(II) removal is 4 h by using nZVI [19]. The importance of contact time comes from the need of identify the possibility of binding and obtaining the optimal time for occupied sites by metal ions and completing removal of the target metal ions. When adsorbent dosage becomes constant in a batch system, number of sites remains constant. After this time, the balance has happened between adsorption and desorption. If the time increases, the removal efficiency does not increase. Then adsorption of Ni(II) and Ag(I) on the iron oxide nanoparticle adsorbent was completed at the 4 hours and it found to be very rapid as evaluated on the basis of time effect.

### Effect of adsorbent dosage on Ni(II) and Ag(I) removal

**Figure 3:**
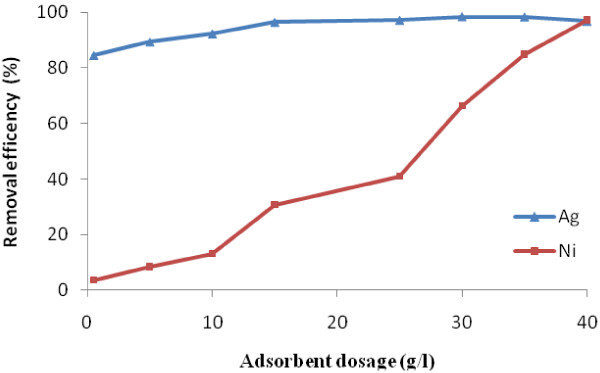
Effect of adsorbent dosage on Ni(II) and Ag(I) removal at pH of 2.7 and t = 4 h.

The effect of iron oxide nanoparticle dosages (0.5, 1, 10, 15, 25, 30, 35, and 40 g/L) at initial pH of 2.7 and 4 h contact time are presented in Figure 3.

It can be seen when the iron oxide nanoparticle dosage increases, the removal efficiency of Ni(II) and Ag(I) increases. There are nearly complete removal (97%) of Ni(II) and Ag(I) at iron oxide nanoparticle dosage of 40 and 20 g/L respectively. Clearly, it`s due to initial contaminant concentration that Ni(II) is 15 but Ag(I) is 5 mg/L. Hu et al. employed magnetic Fe_2_O_3_ nanoparticles as adsorbent for the removal of Cr(VI) from wastewater and the adsorption capacity was found to be very high [20].

Comparison of two graphs (Figure 3) of removal of Ni(II) and Ag(I) in terms of nanoparticle dosage showed a significant difference in removal. The removal of Ni(II) has a nonlinear relationship with the amount of adsorbent, but for Ag(I) the relationship is nearly linear and the high removal was happened in primary time. This difference indicates that the removal mechanism is different for these ions. So that Ni ions adsorb on the surface of the nanoparticle but the Ag ions adsorb on the surface of particles and then reduced by particles core, so removal occurs faster.

### Effect of pH on Ni(II) and Ag(I) removal

**Figure 4:**
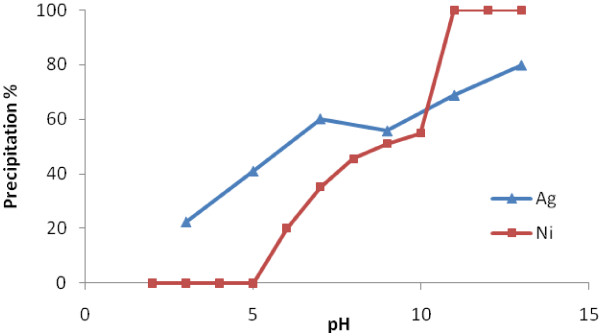
Effect of pH only on Ni(II) and Ag(I) precipitation without adsorbent.

The dependence of adsorption process on pH is an important factor affecting the removal of cations from aqueous solutions [21]. Figure 4 shows the effect of pH only on Ni(II) and Ag(I) removal. Increasing pH leads to metal removal due to precipitation. However when pH increases, precipitation of both metals increases, but precipitation percent of Ag(I) due to increasing pH is less than Ni(II). Ag(I) starts to precipitate at pH=9 but Ni(II) at pH=7.5 theoretically when initial concentration of each metal is 100 mg/L [22]. At pHs more than 10, 96% Ni(II) and 77% Ag(I) precipitates. So the effect of pH on Ni(II) and Ag(I) removal from synthetic nuclear power plant coolant water was assessed at initial pH less than 10.

**Figure 5:**
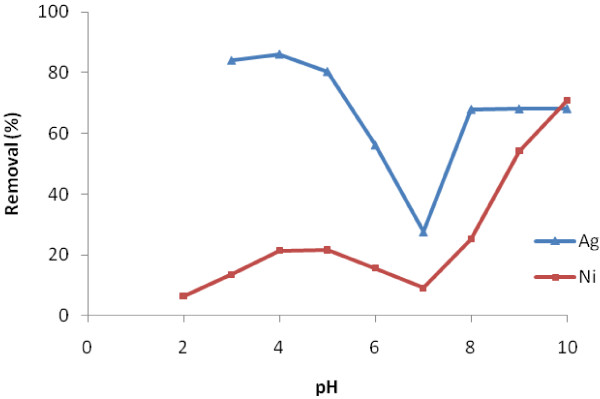
Effect of pH on Ni(II) and Ag(I) removal at 1 g/l adsorbent dosage.

With respect to Ni(II) and Ag(I) precipitation due to change in pH, concentrations of Ni(II) and Ag(I) are determined before adding adsorbent in order not to attribute the removed amount to the precipitation. Figure 5 indicates remaining Ni(II) and Ag(I) removal efficiency at adsorbent of C = 1 g/L, t = 4 h and different pHs. There is the maximum removal efficiency at pH=3 for Ag(I) and at pH=10 for Ni(II). At pH=7, Removal efficiency for both Ni(II) and Ag(I) is minimum. In other words, it is less than pH=6 and 8.

According to Figure 5, Ag(I) removal efficiency in acidic condition is more than alkaline but Ni(II) removal efficiency is more in alkaline condition. It is probably a sign that there is still nZVI besides iron oxide nanoparticle. Because nZVI mechanism for Ag(I) removal is only reduction and removal efficiency by reduction is more in acidic pH. Ni(II) removal efficiency in alkaline pH is more and, it`s common for iron oxide nanoparticle. if there is nZVI, its reduction mechanism for Ni(II) removal is trivial compared to adsorption.

**Figure 6:**
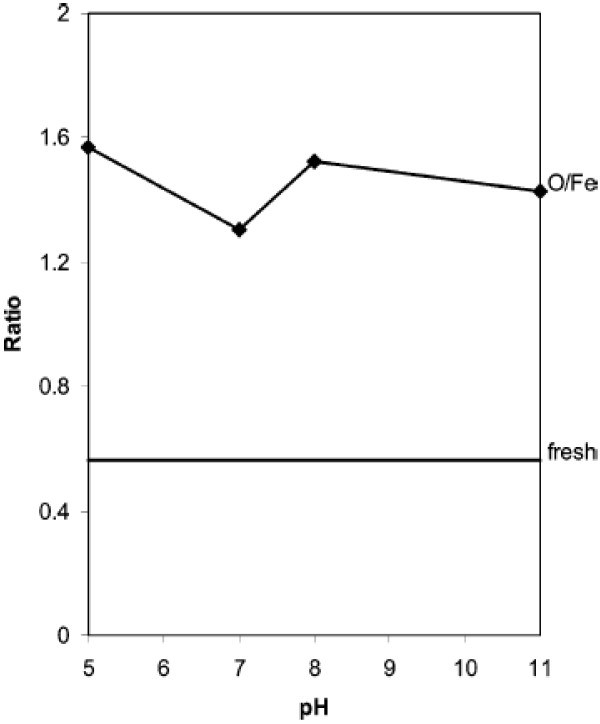
**Photoelectron peak area ratios of total O vs. total Fe **[23]**.**

The trends of elemental ratio as a function of solution pH are depicted in Figure 6. The ratio of O/Fe after 24 h in water increases dramatically compared to that of the fresh nZVI, another indicator of iron oxidation. The XPS survey of iron nanoparticles after 24 h in solutions with pH from 5 to 11 shows that, compared to fresh nZVI, much less Fe(0) is at the surface after 24 h, suggesting continued oxidation of iron. From the peak height and area comparisons, iron oxidation under neutral pH (pH=7 and 8) is less pronounced relative to the more acidic (pH=5) or alkaline (pH=11) conditions [23]. When Figure 5 is compared with photoelectron peak area ratios of total O vs. total Fe (Figure 6) at pH=5-8, there are many similarities between them. In fact, it obeys photoelectron peak area ratios of total O vs. total Fe until pH=8, and increases for pH more than 8 because of adsorption increase.

### Adsorption isotherm study

Analysis of equilibrium data is important for developing an equation that can be used to compare different operational conditions and to design and optimize an operating procedure. The Langmuir and Freundlich equations are commonly used for describing adsorption equilibrium applications. The empirical Freundlich model based on adsorption on a heterogeneous surface is represented by Equation (1) [24]:

(1) $$q_{e}=K_{f}{\left(C_{e}\right)}^{1/n}$$

Where q_e_ is the amount adsorbed at equilibrium (mg/g), C_e_ the equilibrium concentration of the pollutant in the mixture (mg/L), K_f_ and n are equilibrium constants indicative of adsorption capacity and adsorption intensity, respectively. The linear form of Equation (1) is given by relation (2).

(2) $$\ln\;q_{e}=\ln\;K_{f}+\left(1/n\right)\ln C_{e}$$

The Langmuir equation assumes that: (i) the solid surface presents a finite number of identical sites which are energetically uniform; (ii) there is no interaction between adsorbed species, meaning that the amount adsorbed has no influence on the rate of adsorption; (iii) a monolayer is formed when the solid surface reaches saturation. The most widely used Langmuir equation is given by relation (3) [25]:

(3) $$q_{e}=Q^{0}bC_{e}/\left(1+bC_{e}\right)$$

Where q_e_ is the amount adsorbed at equilibrium (mg/g), C_e_ the equilibrium concentration of the pollutant in the mixture (mg/L), b a constant related to the energy or net enthalpy and intensity of adsorption (L/mg), and Q^0^ the mass of adsorbed solute required to saturate a unit mass of adsorbent (mg/g). Q^0^ represents a practical limiting adsorption capacity when the surface is fully covered with metal ions and allows the comparison of adsorption performance, particularly in the cases where the adsorbent did not reach its full saturation in experiments. The linear form of Langmuir model is represented by Equation (4):

(4) $$\mathrm{Ce}/q_{e}=\left(1/Q^{0}b\right)+\left(C_{e}/Q^{0}\right)$$

**Figure 7:**
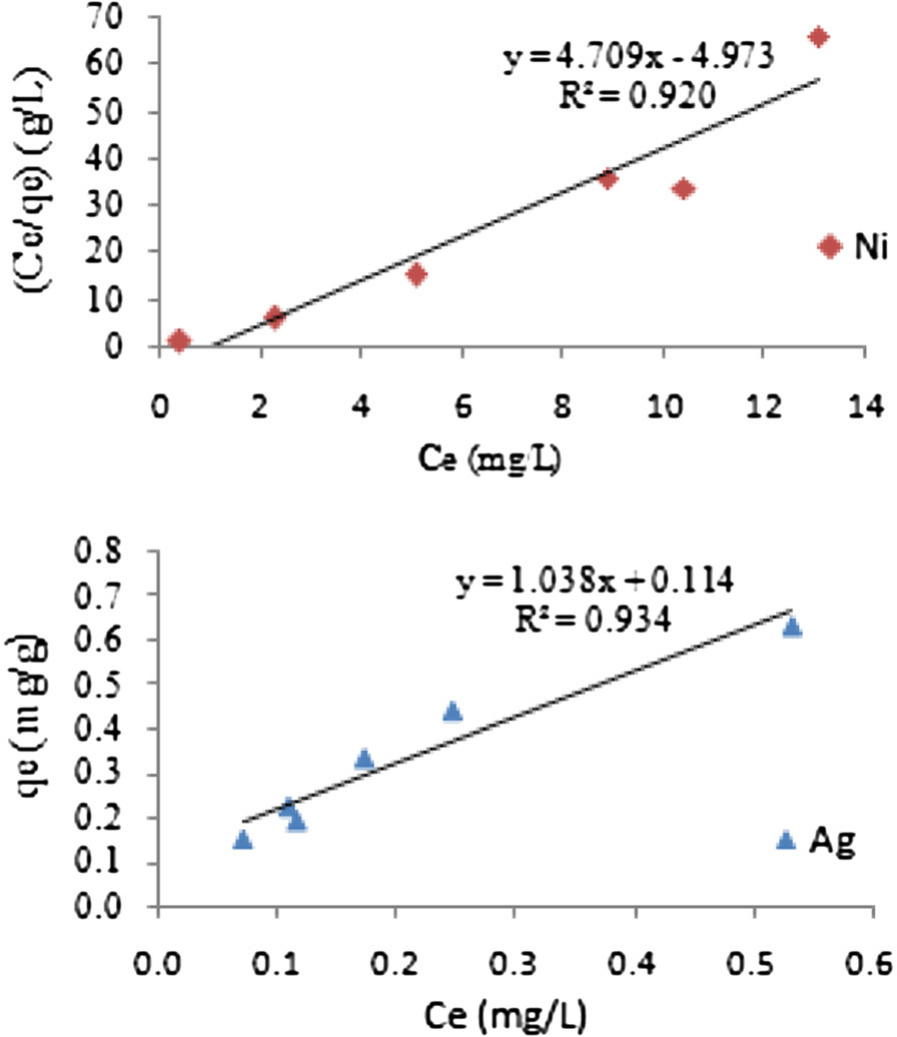
Adsorption isotherms of Ni(II) and Ag(I) on iron oxide nanoparticles.

The equilibrium model in a batch system for Ni(II) and Ag(I) removal was shown in Figure 7. The adsorption constants evaluated from both Langmuir and Freundlich isotherms with the correlation coefficients are given in Table 3. The higher correlation coefficients showed that Langmuir model is more suitable than Freundlich model for describing the adsorption equilibrium of nickel in the studied concentration range and Freundlich model for silver. The Langmuir and Freundlich constants were used to compare the capacity of the iron oxide nanoparticles for adsorption of nickel and silver.

**Table 3:** Freundlich and Langmuir parameters for adsorption of nickel and silver on iron oxide nanoparticles

	**Freundlich constants**			**Langmuir constants**		
	**K**_**f**_	**n**	**R**^**2**^	**Q**^**0**^**(mg/g)**	**b (L/mg)**	**R**^**2**^
Metal						
Ni(II)	-	-	No linear	0.23	−0.04	0.92
Ag(I)	1.30	0.96	0.93	1.24	3.18	0.86

### Adsorption kinetic study

In order to investigate the mechanism of adsorption kinetic models are generally used to test experimental data. Pseudo-first-order and pseudo-second-order equations can be used assuming that the measured concentrations are equal to surface concentrations. The pseudo-second-order kinetic model is expressed as [26,27]:

(5) $$\mathrm{dq}/\mathrm{dt}=k_{2,\mathrm{ads}}{\left(q_{e}–q_{t}\right)}^{2}$$

Where k_2_,_ads_ (g/mg.min) is the rate constant of second-order adsorption. Equation (6) showed the integrated form of Equation (5):

(6) $$1/\left(q_{e}–q_{t}\right)=1/q_{e}+k_{2,\mathrm{ads}}t$$

Equation (6) can be rearranged to obtain equation (7):

(7) $$t/q=1/\left(k_{2,\mathrm{ads}}q_{e}{}^{2}\right)+t/q_{e}$$

**Figure 8:**
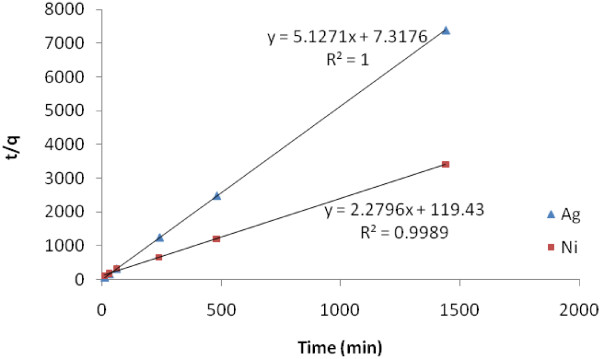
Lagergren plots for the adsorption of Ni(II) and Ag(I) at pH of 2.7 and t = 4 h.

Kinetic plots were carried out to evaluate the best kinetic models. These plots are shown in Figure 8.

The values of different parameters determined from pseudo-second-order kinetic model for the two metal ions along with their corresponding correlation coefficients are presented in Table 4.

**Table 4:** **Adsorption rate constants, q**_**e **_**estimated and correlation coefficients associated to the pseudo-second-order kinetic model**

**Parameter**	**K**_**2,ads**_	**q**_**e **_**(mg/g) Exp.**	**q**_**e **_**(mg/g) Cal.**	**R**^**2**^
Metal	0.04	0.44	0.37	0.99
Ni(II)				
Ag(I)	3.59	0.19	0.19	1.00

The correlation coefficients for the second-order kinetic model are nearly equal to 1 and the theoretical values of q_e_ also agree very well with the experimental values. This suggests that the experiment data of Ni(II) and Ag(I) adsorption on the iron oxide nanoparticles fitted properly with the pseudo-second-order kinetic model. Similar adsorption has been reported such as Pb^2+^ removal from wastewater by water soluble Fe_3_O_4_ nanoparticles as adsorbent in which Langmuir isotherm and pseudo-second-order kinetic model were fitted to experimental Pb^2+^ adsorption and kinetic data [28].

## Conclusion

The behavior of two ions is similar at different adsorbent dosages, time periods, and kinetic model. They are also similar at pH=7and but the removal efficiency trends of two ions at the tested pH range and equilibrium modeling are different. Removal efficiency in alkaline condition is more than acidic pH for Ni(II) and vice versa for Ag(I). Iron oxide nanoparticles have a medium efficiency for removing Ni(II) and Ag(I) from synthetic nuclear power plant coolant water because it`s more efficient at alkaline pH. By evaluating other metals especially Cr(III) and Co(II) removal efficiency, iron oxide nanoparticles can be used to purify synthetic nuclear power plant coolant water.

## Competing interests

The authors declare that they have no competing interests.

## Authors’ contribution

MHS and MAJ participated in the design of the study and draft the manuscript. MAJ carried out the experimental studies. MHE helped to design and draft the manuscript. MA performed statistical analysis of the collected data. All authors read and approved the final manuscript.
